# COVID-19-related violence trend data challenges & a resource for injury researchers

**DOI:** 10.1186/s40621-021-00338-6

**Published:** 2021-07-12

**Authors:** Hannah I. Rochford, Kaleb Brooks, Mark Berg, Cori Peek-Asa

**Affiliations:** 1 Injury Prevention Research Center, University of Iowa, 2190 Westlawn, Iowa City, IA 52242 USA; 2 Department of Health Management and Policy, College of Public Health, University of Iowa, 145 N. Riverside Drive, Room N271, Iowa City, 52242-2007 USA

**Keywords:** Firearm acquisition, Gun violence, Intimate partner violence, Family violence

## Abstract

Published works have raised concerns that certain violent behaviors and firearm acquisition have encountered dramatic increases since the onset of COVID-19. While these works provide important preliminary insights, they lack the empirical robustness necessary to inform a targeted societal response. Having the ability to perform the research needed to support evidence-based policy requires that data at national, state and local-levels be accessible and of sufficient quality. While related, robust data sources do arguably exist, their availability may come long after the window for effective prevention and intervention efforts has closed or may otherwise present with quality limitations, leaving populations at risk for various forms of violence without the support of protective policies.

The University of Iowa Injury Prevention Research Center and the Public Policy Center has compiled a compendium of secondary data sources in an effort to promote exploration of relationships between the COVID-19 pandemic and rates of injury and violence. The forms of violence and firearm-related behavior that were identified as being at risk for amplification given the social stress, economic stress and isolation associated with the public health emergency period included: firearm acquisition, firearm violence, intimate partner violence and family violence.

Access to robust data is critical to support population safety through unprecedented circumstances, like those encountered during COVID-19. During this pandemic many media reports addressed trends related to intimate partner violence (IPV), family violence (FV), and firearm purchases and permits. The University of Iowa Injury Prevention Research and Public Policy Centers have compiled a compendium of secondary data sources in an effort to promote exploration of firearm acquisition and violence related to the COVID-19 pandemic. Our team developed this living resource to (1) promote the uptake of work exploring said relationships and (2) position researchers engaged in this work to select the most appropriate sources of data for their research questions. While this is not a complete list, it covers relevant, frequently used national and local data sources.

## Research resource rationale

Commendable small-scale research efforts and investigatory journalism works cited throughout this work have reported that firearm acquisition and certain types of violence are experiencing upward trends amidst COVID-19. While these reports provide a peek into our new underlying reality, they lack the empirical robustness necessary to guide a societal response. Understanding the underlying truth of COVID-19-related firearm and violence trends will require the field’s continued rigorous analysis of reliable data. However, the complex data collection systems impede both empirical trend estimates, and the societal responses that might manage them (Asher and Horwitz 2020).

The content categories for the compendium (gun ownership, gun violence, IPV, and FV) were selected given (1) the presence of theory and empirical work that support suggested increases, and (2) the journalistic spotlight these outcomes have received. With respect to the former, pandemic-related social isolation and economic pressures are suspected contributors to increases in the prevalence and severity of violence within families and partnerships (Sharma and Borah 2020). These increases have been empirically supported by works such as Jetelina et al. 2020 work finding that physical and sexual IPV intensified early in the pandemic.

With respect to journalism works, the New York Times reported that firearm background checks processed by the FBI reached nearly 2 million in March 2020. This volume was exceeded only following President Obama’s re-election and the Sandy Hook shooting. The relative increase in firearm purchases seems to be most pronounced in areas that have encountered particularly high rates of COVID-19 (Collins and Yaffe-Bellany 2020). USA Today reported that IPV incidents increased by 10–30% in 19 of the 20 police agencies examined from February–March 2020 (Jacoby et al. 2020). Additionally, CNBC reported that a Massachusetts hospital found a significant increase in IPV-related emergency care cases during the first few weeks of the pandemic relative to the cases seen in the corresponding weeks of 2019 (Francis 2020).

## Research resource development & description

The research team used the outcomes of interest as keywords to execute literature searches through Google Scholar, PubMed, and the research team’s institutional library resources to identify relevant secondary data sources. References of the literature identified in this search were also reviewed. Related national advocacy groups who included statistics on their websites were consulted to understand where they derived published figures.

To align identified data and research questions, each compendium entry includes: a data source’s name, oversight authority, the population represented, variables included, the main violence/firearm variable(s), the unit(s) of analysis, demographic variables, a summary of the data’s accessibility and methods of data collection, the frequency with which the data are updated/dates the data are expected to be available, and a small discussion of the data’s unique strengths and limitations.

The compendium is organized by two large sections: data available in real-time (or nearly) and data that is lagged, usually to allow for compilation. Within each section, there are four sub-sections: data related to gun ownership, gun violence, IPV and FV. Within these sub-sections data is categorized by its scope: national or state / local.

Related data sources vary substantively in terms of geographic scope, and in timeliness of their availability. One of the most noteworthy challenges identified in the development of the index was the tension between the demand for real-time data and the long period required to assemble geographically inclusive, comprehensive data sets. Access to real-time information is indispensable to composing and implementing evidence-based responses. However, data that is updated in real-time (or even in the short-term) is less common and generally limited in scope. The limited availability of real-time data is reflected in the data resource.

## Related data challenges

We hope this compendium will aid the field in better understanding the trends and circumstances impacting firearm and interpersonal violence trends during and beyond the COVID-19 pandemic. However, when using this resource, it is critical that challenges inherent to available data be considered. By highlighting these, we hope to position researchers to use our compendium to identify the data best-suited to their research aims.

**Fig. 1:**
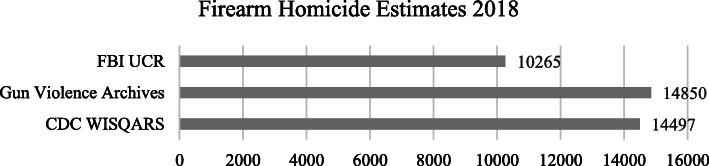
Comparison of annual U.S. firearm homicide estimates for 2018 across secondary data sources

Foremost among these challenges is the variability of case definitions and sample frames, yielding differences in outcome estimates between sources. For example, (as was mentioned previously) the New York Times reported that FBI-processed firearm background checks reached nearly 2 million in March 2020 (Collins and Yaffe-Bellany 2020), while USA Today reported nearly double that figure (estimating 3.7 million background checks were performed by the FBI in March 2020) (Brown 2020). Even amongst some of the most reputable national data sources, different estimates are offered: as shown in Fig. 1, the 2018 estimate of firearm homicides offered by the CDC WISQARS database was 14,497, (National Center for Injury Prevention and Control, CDC NCHS Vital Statistics System for numbers of deaths n.d.) whereas the Gun Violence Archives offers a corresponding estimate of 14,850, (Gun Violence Archive | Past Summary Ledgers 2020) and the FBI UCR’s stood at 10,265 (Expanded Homicide Data Table 8 2020).

While some variation is a function of differences in definition or population scope, others reflect imperfect collection processes. Researchers must spend time understanding differences between data alternatives and how opting for one over another may impact the inferences of their research.

A second crucial challenge is the deficit in representative, national real-time data, which undermines the timely identification of emerging risk factors for violence. For example, some of the best sources of related real-time data are calls-for-service records maintained by police jurisdictions. While timely, these data are limited to single jurisdictions without underlying population data. Further, there is no standardization in what is reported, making compilation of data difficult and comparisons between agencies or time periods questionable (Asher and Horwitz 2020). Limited generalizability is another key challenge in the use of local data.

National data sources are not without their own challenges, however, especially recognizing that they are often compiled from local sources. Their benefits include regularly having standardized case identification and definitions, and the opportunity to use weighting to reflect an underlying population, but these advantages generally entail a compilation lag.

## Conclusion

Empirically sound research requires both the existence and accessibility of data at national, state and local-levels. While some data are available, their value is contingent upon their appropriate use in timely research. Equipped with the compendium and perspectives on data challenges, our team hopes related work by injury and violence researchers will inform policies able to effectively reduce the harms discussed here.

## Data Availability

All data mentioned in this commentary are publicly available and cited to facilitate use by other researchers or interested readers. The data compendium mentioned in the closing section is also publicly available via the included link.

## References

[CR1] Asher J, Horwitz B. Murder rates have increased, but reporting on crime data is still woefully out of date: USA TODAY; 2020, December 29. Retrieved from https://www.usatoday.com/story/opinion/policing/2020/12/29/murder-rates-increased-but-crime-data-woefully-behind-column/4059583001. Accessed 23 May 2021.

[CR2] Brown M. Fact check: guns sales rise and crime falls as the coronavirus spreads in US: USA TODAY; 2020, April 20. Retrieved from https://www.usatoday.com/story/news/factcheck/2020/04/20/fact-check-gun-sales-rise-crime-falls-amid-pandemic/5162481002

[CR3] Collins K, Yaffe-Bellany D. About 2 million guns were sold in the U.S. as virus fears spread: N.Y. Times; 2020, April 1. Retrieved from https://www.nytimes.com/interactive/2020/04/01/business/coronavirus-gun-sales.html

[CR4] Expanded Homicide Data Table 8. (2020, November 29). Crime in the U.S. 2018. FBI: UCR. Retrieved from https://ucr.fbi.gov/crime-in-the-u.s/2018/crime-in-the-u.s.-2018/tables/expanded-homicide-data-table-8.xls

[CR5] Francis S. Op-ed: uptick in domestic violence amid Covid-19 isolation: CNBC; 2020, October 30. Retrieved from https://www.cnbc.com/2020/10/30/uptick-in-domestic-violence-amid-covid-19-isolation.html

[CR6] Gun Violence Archive | Past Summary Ledgers. (2020, November 29). Retrieved from https://www.gunviolencearchive.org/past-tolls. Accessed 23 May 2021.

[CR7] Jacoby K, Stucka M, Phillips K. Crime rates plummet amid the coronavirus pandemic, but not everyone is safer in their home: USA TODAY; 2020, April 4. Retrieved from https://www.usatoday.com/story/news/investigations/2020/04/04/coronavirus-crime-rates-drop-and-domestic-violence-spikes/2939120001

[CR8] Jetelina KK, Knell G, Molsberry RJ (2020). Changes in intimate partner violence during the early stages of the COVID-19 pandemic in the USA. Inj Prev..

[CR9] National Center for Injury Prevention and Control, CDC NCHS Vital Statistics System for numbers of deaths. (n.d.). Homicide/Legal Intervention Firearm Deaths and Rates per 100,000, 2018, Unites States. All Races, Both Sexes, All Ages, ICD-10 Codes: X93-X95, Y35.0, U01.4. Bureau of Census for population estimates. Retrieved from https://webappa.cdc.gov/cgi-bin/broker.exe. Accessed 23 May 2021.

[CR10] Sharma A, Borah SB. Covid-19 and domestic violence: an indirect path to social and economic crisis. J Fam Violence. 2020:1–7. 10.1007/s10896-020-00188-8. 10.1007/s10896-020-00188-8 PMC7386835 32836737

